# Towards *In Vivo* Imaging of Cancer Sialylation

**DOI:** 10.1155/2011/283497

**Published:** 2011-09-19

**Authors:** Ivan Martinez-Duncker, Roberta Salinas-Marin, Carlos Martinez-Duncker

**Affiliations:** ^1^Human Glycobiology Laboratory, Science Faculty, Autonomous University of the State of Morelos, Avenida Universidad 1001, Col. Chamilpa, 62209 Cuernavaca, MOR, Mexico; ^2^The Molecular Imaging Commission of the Mexican Academy of Surgery, Zacatecas 230-502, Colonia Roma, 06700 Mexico, DF, Mexico

## Abstract

*In vivo* assessment of tumor glucose catabolism by positron emission tomography (PET) has become a highly valued study in the medical management of cancer. Emerging technologies offer the potential to evaluate *in vivo* another aspect of cancer carbohydrate metabolism related to the increased anabolic use of monosaccharides like sialic acid (Sia). Sia is used for the synthesis of sialylated oligosaccharides in the cell surface that in cancer cells are overexpressed and positively associated to malignancy and worse prognosis because of their role in invasion and metastasis. This paper addresses the key points of the different strategies that have been developed to image Sia expression *in vivo* and the perspectives to translate it from the bench to the bedside where it would offer the clinician highly valued complementary information on cancer carbohydrate metabolism that is currently unavailable *in vivo*.

## 1. Introduction

There is no doubt that *in vivo* imaging of glucose catabolism by positron emission tomography (PET) through the use of the PET tracer Fluorine-18-2-*fluoro*-2-deoxy-D-glucose (FDG) has revolutionized diagnosis and staging of cancer [1]. FDG, an analogue of glucose, is transported into cells by glucose transporters and follows the glycolytic pathway being phosphorylated by hexokinase into FDG-6-phosphate. At this point FDG is metabolically trapped because it cannot follow the normal glycolytic pathway due to the substitution of the 2′ hydroxyl group by Fluorine 18 (^18^F), causing its accumulation in the cell. Higher accumulation of FDG in cancer cells compared to normal cells allows the imaging by PET of the “Warburg effect” or aerobic glycolysis which is uniquely observed in cancer [2]. The association of this effect to malignancy has made FDG an indispensable or at least a very important imaging agent in the diagnosis, staging, restaging, and assessment of treatment in various types of cancer including lung, colorectal, esophageal, stomach, head and neck, thyroid, cervical, ovarian, and breast cancers, as well as melanoma and most types of lymphoma [1, 3].

The *in vivo* imaging of anabolic rather than catabolic carbohydrate pathways has a potential usefulness in cancer management by offering the clinician complementary information on cancer carbohydrate metabolism that is currently unavailable *in vivo*. This paper addresses the key points of different strategies that have been developed to image the *in vivo* expression of sialylated oligosaccharides synthesized by an essential cellular process known as glycosylation [4]. Glycosylation involves the synthesis and attachment to proteins (glycoproteins) and lipids (glycolipids) of a structurally diverse group of oligosaccharides chains also known as glycans that are synthesized in the endoplasmic reticulum and Golgi apparatus of all human cells. Along this paper the role of sialylated antigens in the glycophenotype of cancer, their biosynthesis, and emerging imaging tools are described in text and figures as the foundations of a preliminary suggestion towards a new clinical approach in tumor imaging.

Each type of human cell displays in its surface an array of glycans attached to proteins or lipids to form glycoconjugates. The different types of monosaccharides and linkage combinations can be found on glycans code for biological information that translates into structural or functional properties of the glycoconjugate. The type and degree of expression of glycans is determined by the type and activities of the proteins involved in their synthesis that can be different from one cell type to another and even differ to some extent between the same types of cell. This dynamic temporospatial characteristic of glycosylation and the great intrinsic information it can carry as a “glycan code” have been evolutionarily selected as an important mechanism for cells to communicate and enhance distinct physiologic and pathologic states, including malignant transformation [5].

The “glycan code” has been probed and imaged extensively *in vitro* mostly by the use of antibodies that recognize carbohydrate epitopes or through carbohydrate-binding proteins known as lectins [6]. In the case of cancer certain characteristics of the glycan code have been clearly associated to increased tumor aggressiveness and worse prognosis. One of these being the overexpression of sialic acid (Sia) residues that are present in many characterized carbohydrate antigens [7]. 

## 2. Sialylated Antigens in the Glycophenotype of Cancer

**Figure 1:**
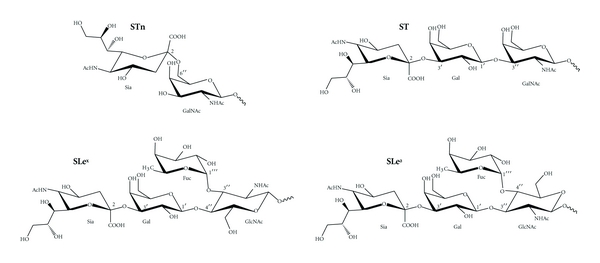
Chemical structures of sialylated antigens associated to malignancy. Sialyl-Tn (Sia*α*2→6GalNAc-T/S), sialyl T (Sia*α*2-3Gal*β*1-3GalNAc-T/S), sialyl Lewis X (SLe^x^; Sia*α*2,3Gal*β*1,4(Fuc*α*1,3)GlcNAc), and sialyl Lewis A (SLe^a^; Sia*α*2,3Gal*β*1,3(Fuc*α*1,4)GlcNAc).

A substantial component that distinguishes malignant from benign glycophenotypes is the neoexpression or overexpression of sialylated epitopes in the cell surface that include sialyl-Tn (STn; Sia*α*2→6GalNAc-T/S), sialyl T (ST; Sia*α*2-3Gal*β*1-3GalNAc-T/S), sialyl Lewis X (SLe^x^; Sia*α*2,3Gal*β*1,4(Fuc*α*1,3)GlcNAc), and sialyl Lewis A (SLe^a^/CA19-9; Sia2,3Gal*β*1,3(Fuc*α*1,4)GlcNAc), Figure 1. Additionally, various tumors including small cell and nonsmall cell lung carcinomas, multiple myeloma, neuroblastoma, and Wilms' tumor express Sia as a homopolymer called polysialic acid or PSA (Sia*α*2,8Sia) [8–12]. The neo- or overexpression of these sialylated antigens is mainly caused by an increase in the expression of sialyltransferases (STs) and fucosyltransferases (FUTs), glycosyltransferase enzymes responsible for their synthesis that add Sia and fucose residues to glycans, respectively. Additionally, effective increase in antigen synthesis requires not only glycosyltransferase overexpression but also adequate donor and acceptor substrate synthesis which requires regulation of other elements of the glycosylation machinery [13]. This increase in sialylated antigen expression during cancer progression has been associated to hypoxic conditions that develop in a growing tumor and induce transcription of STs, FUTs, and nucleotide-sugar transporters that provide them with donor substrate [14–17].

Overexpression of sialylated antigens like SLe^a^ and SLe^x^ plays an important role in the biology of cancer, particularly the metastatic process. Overexpression of these antigens in circulating cancer cells allows them to attach to a family of endogenous lectins called selectins that are expressed in endothelial cells of high endothelial venules (HEVs), but also platelets and leukocytes [18]. Selectins normally participate in the first phase of what is known as the leukocyte adhesion cascade that allows leukocytes to roll over the inflamed vascular endothelium through selectin-carbohydrate interactions mediated by ligands that express SLe^x^ and SLe^a^ epitopes. Leukocyte rolling gives way to more stable interactions with endothelial integrins that allow them to transmigrate to the interstitial space to culminate an immune response [18]. Cancer cells that disperse from a primary tumor and access the vascular compartment have exploited this leukocyte adhesion cascade mechanism to attach to HEV and extravasate to initiate the establishment of metastatic lesions. Also, overexpression of SLe^a/x^ antigens has been found to facilitate tumor angiogenesis by mediating cancer cell adhesion to endothelial cells [19, 20].

It is clear that increased expression of sialylated antigens on cancer cells is closely implicated in the process of cancer progression, and more malignant cancer cells tend to have a more enhanced expression of these carbohydrate determinants [19]. The numerous clinical statistics made available to date show that the intensity of SLe^a/x^ and sialyl Tn expression on cancer cells significantly correlates with the prognosis of patients and are reviewed elsewhere [21–24]. A statistically significant correlation between the postoperative patient prognosis and SLe^a^ expression has been reported for colon and stomach cancers while its correlation with SLe^x^ expression has been reported for lung, breast, prostate, stomach, colon, and urinary bladder cancers [25]. Also, in both small cell and nonsmall cell lung carcinomas, colorectal cancer, and multiple myeloma, the expression of PSA has been correlated with tumor progression [8, 9, 11, 12, 26]. Unfortunately, the determination of the sialylation status, particularly of solid tumors, is currently performed mainly on tissue sections obtained from tumor biopsies limiting the number of possible assessments through time.

## 3. Sialic Acid and Its Biosynthesis

**Figure 2:**
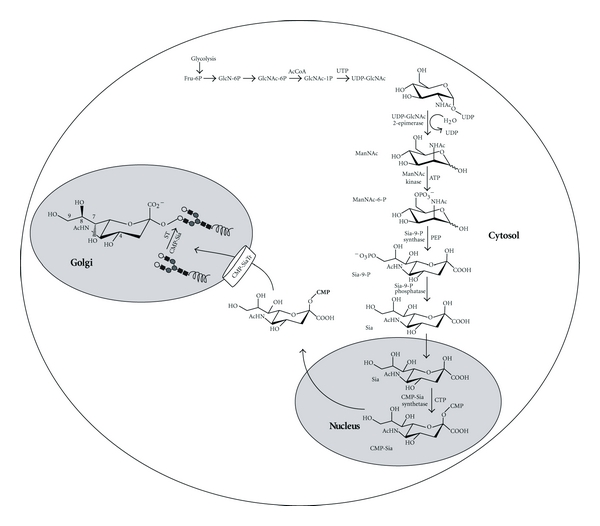
The biosynthesis of CMP-Sia in mammals. Sialic acid (Sia) is a nonulosonic amino sugar with a carboxylate at the C1 position that is ionized at physiological pH giving it a negative charge. Sia is synthesized in the cytosol and is activated in the form of CMP-Sia in the nucleus. The CMP-Sia is transported to the cytosol by an unknown mechanism and subsequently to the Golgi lumen by the CMP-Sia transporter (CMP-SiaTr) where it serves as a donor substrate to sialyltransferases (STs) that incorporate it at the nonreducing end of mature glycans. CMP: cytidine monophosphate.

The name sialic acid collectively refers to a family of over 50 naturally occurring sugars and a growing number of synthetic analogs. In humans the predominant Sia is *N*-acetylneuraminic acid [27]. Sia is a nonulosonic amino sugar with a carboxylate at the C1 position that is ionized at physiological pH giving it a negative charge (Figure 2). Sia has the potential for additional substitutions with acetyl, methyl, sulphate, and phosphate groups at the hydroxyl groups on the 4-, 7-, 8-, and 9-carbons that give it additional properties. The negative charge of Sia and its terminal position in glycans have given it a predominant role in determining the nature of glycan interactions involved in many essential functions of human physiology. 

The biosynthesis of Sia begins in the cytosol with the formation of *N*-acetylmannosamine (ManNAc) from a relatively minor proportion of UDP-*N*-acetylglucosamine (UDP-GlcNAc), obtained form the extracellular environment [28] or derived from GlcNAc via the action of GlcNAc 2-epimerase [29] (Figure 2). In mammals, the ManNAc is then phosphorylated to give ManNAc-6-phosphate (ManNAc-6P). The second step involves the condensation of either ManNAc or MacNAc-6P with phosphoenolpyruvate (PEP) to give neuraminic acid or neuraminic acid-9P, respectively. In mammals, neuraminic acid-9P is then dephosphorylated to generate neuraminic acid (Sia). Finally, in the nucleus the activation of Sia with cytidine monophosphate (CMP-Sia) is generated with the use of cytosine triphosphate (CTP) [30]. Once in the cytosol CMP-Sia is then transported to the Golgi lumen by a specific transporter (CMP-SiaTr) where it is used as donor substrate by more than twenty human sialyltransferases (STs) that incorporate it into the nonreducing end of glycans [16, 31, 32].

**Figure 3:**
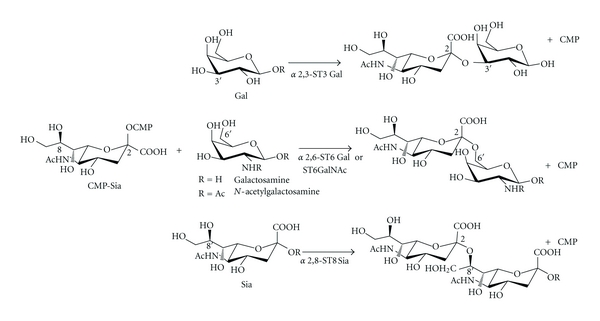
Types of sialyltransferases and their main acceptor substrates. STs can link sialic acid (Sia) to terminal galactose (Gal) via *α*2,6 or *α*2,3-linkage or to galactosamine or *N*-acetyl-galactosamine (GalNAc) via *α*2,6-linkage. Also, Sia can be linked to the C8 position of another Sia residue forming polysialic acids. CMP: cytidine monophosphate.

Linkage of Sia by STs can be done to a terminal galactose residue (Gal) via *α*2,6 or *α*2,3-linkage, to *N*-acetyl-galactosamine (GalNAc) or galactosamine residues via *α*2,6-linkage, or to another Sia as an *α*2,8 homopolymer (Figure 3). The presence or absence of Sia as well as the type of linkage it presents in the glycan have been selected in evolution as recognition characteristics that allow or hamper biding of glycan ligands, thus determining important molecular interactions that affect cell behavior including cell-cell, cell-matrix, and cell-soluble molecule interactions that play roles in both physiological and pathological processes [7].

## 4. Sialic Acid Imaging* In Vivo*

Because of the clear association that has been established between sialylated antigen overexpression and tumor aggressiveness, the imaging of tumor sialylation would offer a concomitant or alternative study to PET-FDG imaging not only to diagnose cancer but also to assess patient's prognosis. To obtain the *in vivo* sialylation status of a tumor is a clinical asset that must be translated from the bench to the bedside. There are at least two strategies for the *in vivo* imaging of tumor Sia expression. One involves metabolic labeling that allows imaging of *de novo* synthesis of Sia, and the second one involves native Sia recognition by imaging probes.

### 4.1. Metabolic Labeling

Metabolic labeling allows the study of a metabolic pathway by administering to the cells compounds that are modified analogs of natural substrates for that particular pathway. Modification of these compounds aims at providing a means for their detection without interfering with their metabolic use. An ideal candidate compound for evaluating tumor sialylation *in vivo* has to be cell permeable, has to be able to enter the CMP-Sia biosynthesis pathway at a committed stage, and although modified has to be efficiently used by STs. The use of labelled Sia or CMP-Sia is restricted because of the lack of plasma membrane transporters and the negative charge of unmodified Sia that limits its cellular permeability. Uptake studies with ^18^F-labelled sialic acids *N*-acetyl-3-[^18^F]fluorosialic acid (3-Sia) and *N*-acetyl-2-deoxy-2,3-difluorosialic acid (2,3-diSia) showed inefficient uptake and unsuitability for *in vivo* imaging [33]. An alternative to solve this problem is the use of modified permeable Sia analogs [34, 35] or permeable Sia precursors that can be subsequently detected by imaging probes. Sia precursors, particularly based on ManNAc, have been favored over Sia analogs for *in vitro* and *in vivo* imaging because of lower cost and synthetic tractability of ManNAc over Sia [36].

Of the different analogue precursors of Sia biosynthesis, ManNAc analogues are ideal candidates for metabolic labeling because ManNAc is the first pathway intermediate to be committed to Sia biosynthesis, assuring that imaging of the labelled ManNAc analog reflects glycan Sia expression. And although ManNAc is permeable, ManNAc analogs can be further acetylated to increase passive membrane permeability [37].

Among many modifications that have been achieved on the ManNAc sugar with chemical reporters [38] that allow its detection, only an azide-labelled bioorthogonal chemistry [39] has successfully been used to image *in vivo* its incorporation as glycan-bound Sia [40]. The azide group at the C2 carbon of ManNAc analogs does not interfere with the flux of the precursor towards Sia synthesis, activation in the form of CMP-Sia or ST-mediated transfer. Also, it is abiotic allowing the development of both intracellular and surface chemoselective ligation strategies for its efficient detection by bioorthogonal chemistry through modified phosphines or alkynes.

**Figure 4:**
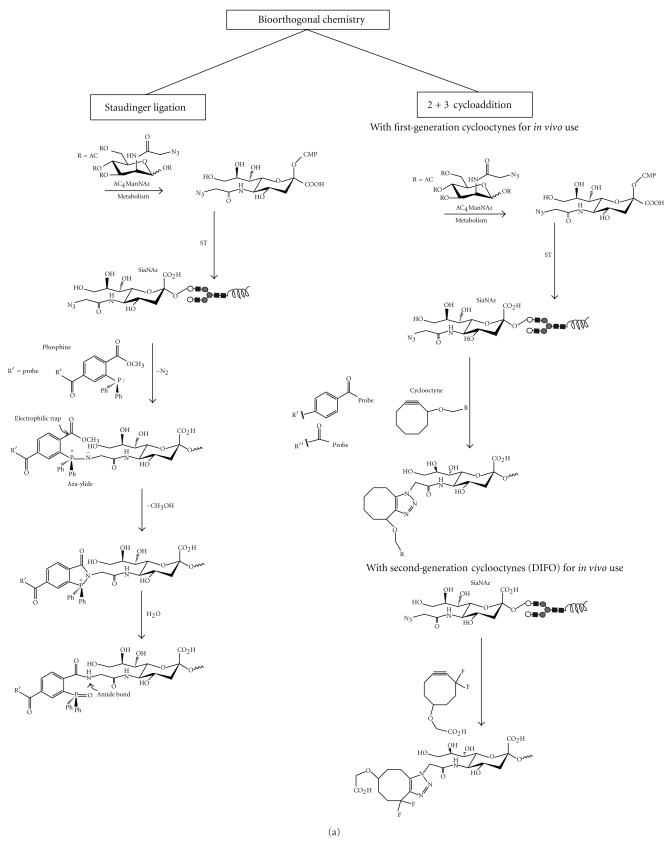

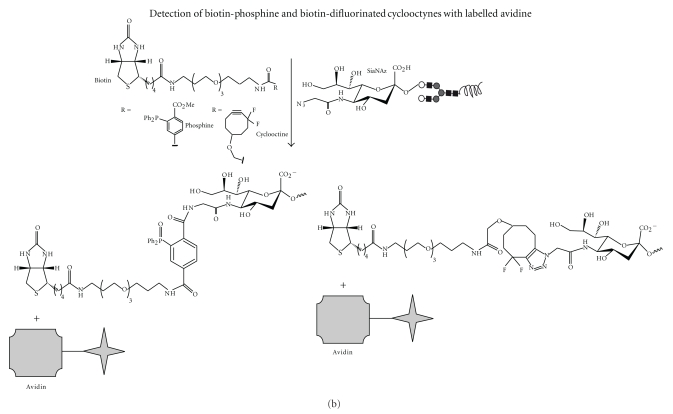
Reactions in bioorthogonal chemistry for detection of azide-modified sialic acid. (a) Two reactions involving bioorthogonal chemistry of azide-modified sialic acid (Sia) have been developed for *in vivo imaging*, the strain-promoted [3 + 2] cycloaddition, and the Staudinger ligation. In both cases a peracetylated analogue of *N*-acetylmannosamine (Ac4ManNAz) enters cells by enhanced passive diffusion and is deacetylated by intracellular carboxylesterases. The resulting ManNAz molecule is then converted to *N*-azidoacetyl sialic acid (SiaNAz) in the cytosol and transported to the nucleus, where it is activated with cytidine monophosphate (CMP) to form CMP-SiaNAz. CMP-SiaNAz is then incorporated into glycans as SiaNAz by the action of STs in the Golgi. The Staudinger ligation allows azide group detection of SiaNAz with a phosphine-substituted ester. The harmful byproduct nucleophilic aza-ylide is captured by intramolecular cyclization by an electrophilic trap (methyl ester) within the phosphine structure allowing it to be used in living animals without physiological harm because phosphine oxide is not produced. Regarding the strain-promoted [3 + 2] cycloaddition of azides the cyclooctyne molecule possesses a ring strain and electron-withdrawing fluorine substituents that together promote the [3 + 2] dipolar cycloaddition with azides to form regioisomeric mixtures of triazoles. This reaction occurs more rapidly than the Staudinger ligation, can be used *in vivo,* and does not require auxiliary reagents. First- and second-generation cyclooctyne (DIFO) compounds have been used in [3 + 2] cycloaddition. (b) Modification of phosphines or cyclooctynes with biotin and subsequent detection with fluorescent or radioisotope labelled avidins have been used for SiaNAz detection *in vivo* in animal models of lung cancer, a similar strategy could be employed using cyclooctynes.

A peracetylated analogue of ManNAc bearing the azide chemical reporter group (Ac4ManNAz) has been administered to mice *in vivo* without toxicity and was found to be suitable for imaging [41, 42]. After cellular uptake Ac4ManNAz is deacetylated in the cytoplasm and then metabolically converted to *N*-azidoacetyl sialic acid (SiaNAz) that is then activated with CMP and used by STs as donor substrate to be incorporated into glycans ( Figure 4(a)). The glycan-associated SiaNAz is then probed *in vivo* through the “Staudinger ligation” that allows the azide group to interact with triphenylphosphine derivatives [39]. An electrophilic trap of methyl ester group in ortho position to the phosphine structure provides a reactive site for the nucleophilic nitrogen from the azide group that temporarily forms an aza-ylide intermediary (not stable in water) with the phosphorus atom. Intramolecular cyclization between the nitrogen and the carbonyl group of the methyl ester group releases methanol and forms a stable amide bond rather than the products of aza-ylide hydrolysis. 

*In vivo* animal imaging potential of this strategy in a murine model of lung carcinoma has been recently reported [43]. Ac_4_ManNAz was pulsed in mice intraperitoneally for 7 days. After this labeling period the resulting SiaNAz was detected by administration on day 11 of biotinylated phosphines (Figure 4(b)) that were detected 2 hours after by intravenous injections of fluorescent or radionuclide-labelled avidin molecules. Radionuclide detection with a SPECT camera which is the most closely related to what human applications would use was achieved with an Indium-111-labelled Neutravidin-DOTA, showing a significant azido-labelled ManNAc-dependent increase in tumor-to-tissue contrast. As the authors of this paper suggest, this strategy requires further improvement by reducing it to a one-step probe approach by using phosphine molecules modified with gamma or positron emitting radioisotopes and with an improved hydrophilic nature of phosphines to reduce the nonspecific stromal binding that was observed in this study. Also, other compounds like cyclooctynes could be used to detect azide groups via strain-promoted azide-alkyne cycloaddition (Figure 4(a)) [44]. The cyclooctyne molecule can be more easily modified to generate reactants with enhanced kinetics. Some derivates possess a ring strain and electron-withdrawing fluorine substituents that together promote the [3 + 2] dipolar cycloaddition with azides to form regioisomeric mixtures of triazoles. This reaction has higher reaction kinetics compared to the Staudinger ligation [38] and has been further improved by synthesizing second-generation difluorinated cyclooctynes (DIFOs) that have propargylic fluorine atoms that increase its interaction energy with the azide (Figure 4(a)) [45]. These DIFOs have been successfully employed for dynamic *in vivo *imaging of developing zebrafish [41], and important advances have been made to improve their synthesis [46].

It is worth considering that fluorophore modified phosphines or cyclooctynes could also be used for imaging of the sialylation status of superficial cancers like skin melanoma or colorectal cancer through fluorescent endoscopy. It is noteworthy to mention that advances in positron emitters through the Staudinger ligation have been achieved using phosphine-substituted thioesters with ^18^F-fluoroethylazide [47].

### 4.2. Probes for Recognition of Native Sia

A second strategy consists in using imaging probes that recognize native Sia in the cell surface. Historically, sialic acids have been explored *in vitro* using plant lectins like wheat germ agglutinin (WGA) that detects Sia independently of its type of linkage, *Sambucus nigra* lectin (SNA) that recognizes *α*2,6 Sia or *Maackia amurensis* aggluttinin (MAL) that recognizes *α*2,3 Sia. Also, antibodies directed against SLe^x^ or SLe^a^ determinants have been used for many years; this is the case of CA19-9 a tumoral marker used for detection of SLe^a^ determinants in gastrointestinal tumors. These *in vitro *tools have been difficult to translate *in vivo* not only because of their immunogenicity, that at least for antibodies could be circumvented by humanizing them, but also because of their molecular weight, that would restrict them to the vascular compartment. This vascular restriction would not be a problem if the aim was to image the expression of Sia on the vascular network of a tumor, for example, or in chronic inflammatory diseases. In the case of lectins, immunogenicity could be avoided if human endogenous sialic-acid-recognizing lectins were to be used like siglecs or selectins, but again their use would probably be limited to the vascular compartment.

**Figure 5:**
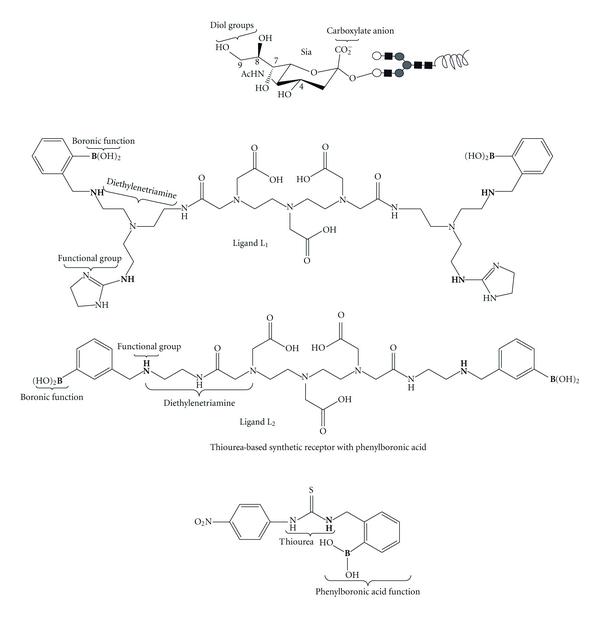
Receptors for native recognition of Sia. The carboxylate and diol groups of sialic acid have been exploited for recognition purposes by lanthanide ligand DTPA-bisamide compounds L1 (3,9-Bis{6-[(4,5-dihydroimidazol-2-yl)aminoethyl]-10-[2-(dihydroxyboranylphenyl)]-2-oxo-3,6,9-triazadecyl}-6-carboxymethyl-,6,9-triazaundecanedioic acid) and L2 (3,9-Bis[3-(dihydroxyboranylphenyl)-2-oxo-3,6-diazaheptyl]-6-carboxymethyl-3,6,9-triazaundecanedioic acid) bearing boronic and functional groups (L1: aminoimidazolium and L2: aminomethyl). Also, a thiourea-based synthetic receptor (2-{[3-(4-Nitrophenyl)ureido]methyl}phenylboronic acid) has been reported to show a high specificity towards Sia.

Nonetheless, other alternatives show promise mainly in the field of magnetic resonance imaging for the detection of native Sia molecules (Figure 5). Frullano et al. reported the synthesis of two lanthanide ion ligands denominated L1 (3,9-Bis{6-[(4,5-dihydroimidazol-2-yl)aminoethyl]-10-[2-(dihydroxyboranylphenyl)]-2-oxo-3,6,9-triazadecyl}-6-carboxymethyl-,6,9-triazaundecanedioic Acid) and L2 (3,9-Bis[3-(dihydroxyboranylphenyl)-2-oxo-3,6-diazaheptyl]-6-carboxymethyl-3,6,9-triazaundecanedioic acid). These compounds were obtained by inclusion of a central chelating unit based on the DTPA-bisamide structure (DTPA, diethyl-enetriamine pentaacetic acid). Molecular modeling studies indicate that both compounds can allow two-site binding to Sia through ester formation by interaction of the boronate function in the ligand with the geminal diol of Sia and an electrostatic interaction between a positively charged aminoimidazolium (L1) or aminomethyl (L2) group present in metaposition relative to the boronic function and the carboxylate group of Sia [48]. Both gadolinium complexed ligands showed high specific and reversible binding towards Sia. The dynamic equilibrium that the compounds showed between their sialic-acid-bound and free states would allow them to be excreted physiologically. Similarly, a thiourea-based synthetic receptor (2-{[3-(4-Nitrophenyl)ureido]methyl}phenylboronic acid) reported by Regueiro-Figueroa et al. that contains phenylboronic acid functions also showed a high specificity towards Sia due to cooperative binding through ester formation with the phenylboronic acid and hydrogen bond interactions between the carboxylate group and the thiourea moiety (Figure 5) [49].

This native recognition approach compared to metabolic labelling has the potential to recognize the type of Sia linkages and even carbohydrate antigens, offering additional specific information, but this aim requires further research.

## 5. Conclusions

Although glycans equip the cell surface with efficient and dynamic signalling mechanisms that have been partially deciphered to convey important clinical information, glycans and glycan-mediated interactions have been rarely targeted for *in vivo* imaging with some exceptions that particularly involve quantification of liver function [50–52].

*In vivo* imaging of tumor sialylation is in the transition stage from the bench to the bedside and is only limited by minor improvements in radioisotope probe synthesis to be used concomitantly or alternatively with FDG in the medical management of cancer. The metabolic labelling with Sia precursor analogs and detection offered by bioorthogonal chemistry offer at this moment a high value for clinical use in the near future. It is important to press for the continuing technological development of these strategies to assess as soon as possible their clinical value in diagnosis, staging, prognosis, and treatment response. Imaging of tumor sialylation brings together information of both catabolic and anabolic processes associated to malignancy which can only improve patient management.
